# Supplementary material for: Sunburn, Sun Safety and Indoor Tanning Among Schoolchildren in Ireland
Source: Int J Public Health. 2021 May 20;66:1604045. doi: 10.3389/ijph.2021.1604045 (PMC8284860; doi:10.3389/ijph.2021.1604045)
Supplement: Supplementary file 2 [file DataSheet1.PDF]

Sunburn, sun safety and indoor tanning among schoolchildren in Ireland

Supplementary tables

Table S1. Using a hat on sunny days across gender in the 2018 Health Behaviour in School-aged Children study in Ireland (*n* = 10,032)

	**Boys**	**Girls**
**Always**	5.5% (258)	2.8% (152)
**Sometimes**	45.1% (2116)	41.5% (2215)
**Never**	49.4% (2319)	55.7% (2972)

Table S2. Using a hat on sunny days across age in the 2018 Health Behaviour in School-aged Children study in Ireland (*n* = 10,032)

	**10-11 years**	**12-14 years**	**15-17 years**
**Always**	7.0% (122)	3.6% (178)	3.3% (110)
**Sometimes**	56.5% (987)	42.4% (2082)	37.4% (1262)
**Never**	36.5% (638)	54.0% (2649)	59.4% (2004)

Table S3. Using a hat on sunny days across social class in the 2018 Health Behaviour in School-aged Children study in Ireland (*n* = 280)

	**SC1-2**	**SC3-4**	**SC5-6**
**Always**	3.9% (213)	4.4% (155)	3.9% (42)
**Sometimes**	44.7% (2421)	42.1% (1492)	39.1% (418)
**Never**	51.4% (2785)	53.5% (1896)	57.0% (610)

Table S4. Wearing sunglasses on sunny days across gender in the 2018 Health Behaviour in School-aged Children study in Ireland (*n* = 10,094)

	**Boys**	**Girls**
**Always**	8.0% (378)	20.7% (1118)
**Sometimes**	52.1% (2451)	62.8% (3385)
**Never**	39.8% (1874)	16.5% (888)

Table S5. Wearing sunglasses on sunny days across age in the 2018 Health Behaviour in School-aged Children study in Ireland (*n* = 10,094)

	**10-11 years**	**12-14 years**	**15-17 years**
**Always**	13.5% (235)	14.4% (710)	16.2% (551)
**Sometimes**	60.5% (1056)	57.0% (2817)	57.6% (1963)
**Never**	26.1% (455)	28.6% (1413)	26.2% (894)

Table S6. Wearing sunglasses on sunny days across social class in the 2018 Health Behaviour in School-aged Children study in Ireland (*n* = 10,094)

	**SC1-2**	**SC3-4**	**SC5-6**
**Always**	14.4% (789)	15.1% (15.1%)	15.8% (169)
**Sometimes**	57.9% (3161)	58.5% (58.5%)	55.4% (594)
**Never**	27.7% (1512)	26.4% (940)	28.9% (310)

Table S7. Wear clothes that cover arms and legs on a sunny day across gender in the 2018 Health Behaviour in School-aged Children study in Ireland (*n* = 10,072)

	**Boys**	**Girls**
**Always**	6.8% (318)	5.6% (302)
**Sometimes**	42.1% (1976)	45.2% (2428)
**Never**	51.1% (2401)	49.2% (2647)

Table S8. Wear clothes that cover arms and legs on a sunny day across age in the 2018 Health Behaviour in School-aged Children study in Ireland (*n* = 10,072)

	**10-11 years**	**12-14 years**	**15-17 years**
**Always**	4.6% (81)	6.4% (313)	6.7% (226)
**Sometimes**	39.7% (693)	42.2% (2079)	48.0% (1632)
**Never**	55.7% (973)	51.5% (2536)	45.3% (1539)

Table S9. Wear clothes that cover arms and legs on a sunny day across social class in the 2018 Health Behaviour in School-aged Children study in Ireland (*n* = 10,072)

	**SC1-2**	**SC3-4**	**SC5-6**
**Always**	5.7% (310)	6.3% (222)	8.2% (88)
**Sometimes**	43.4% (2365)	42.9% (1520)	48.2% (519)
**Never**	50.9% (2775)	50.9% (1804)	43.6% (469)

Table S10. Avoiding the sun between 12 and 3pm on sunny days across gender in the 2018 Health Behaviour in School-aged Children study in Ireland (*n* = 10,058)

	**Boys**	**Girls**
**Always**	3.1% (145)	3.6% (193)
**Sometimes**	24.5% (1152)	33.2% (1783)
**Never**	72.4% (3396)	63.2% (3389)

Table S11. Avoiding the sun between 12 and 3pm on sunny days across age in the 2018 Health Behaviour in School-aged Children study in Ireland (*n* = 10,058)

	**10-11 years**	**12-14 years**	**15-17 years**
**Always**	4.4% (77)	3.4% (167)	2.8% (94)
**Sometimes**	34.4% (600)	29.5% (1449)	26.1% (886)
**Never**	61.1% (1065)	67.1% (3300)	71.2% (2420)

Table S12. Avoiding the sun between 12 and 3pm on sunny days across social class in the 2018 Health Behaviour in School-aged Children study in Ireland (*n* = 10,058)

	**SC1-2**	**SC3-4**	**SC5-6**
**Always**	3.4% (184)	3.0% (108)	4.3% (46)
**Sometimes**	28.2% (1535)	29.7% (1053)	32.5% (347)
**Never**	68.4% (3725)	67.3% (2386)	63.2% (674)

Table S13. Using sunscreen on sunny days across gender in the 2018 Health Behaviour in School-aged Children study in Ireland (*n* = 10,179)

	**Boys**	**Girls**
**Always**	22.8% (1083)	38.1% (2067)
**Sometimes**	54.1% (2569)	49.3% (2679)
**Never**	23.1% (1095)	12.6% (686)

Table S14. Using sunscreen on sunny days across age in the 2018 Health Behaviour in School-aged Children study in Ireland (*n* = 10,179)

	**10-11 years**	**12-14 years**	**15-17 years**
**Always**	45.6% (811)	31.8% (1585)	22.1% (754)
**Sometimes**	45.7% (814)	50.5% (2518)	56.1% (1916)
**Never**	8.7% (155)	17.7% (882)	21.8% (744)

Table S15. Using sunscreen on sunny days across social class in the 2018 Health Behaviour in School-aged Children study in Ireland (*n* = 10,179)

	**SC1-2**	**SC3-4**	**SC5-6**
**Always**	32.0% (1761)	30.7% (1101)	26.6% (288)
**Sometimes**	52.2% (2876)	51.5% (1848)	48.4% (524)
**Never**	15.8% (872)	17.8% (639)	25.0% (270)

[a] Cleaned for a combination of 3, 4, or 5 positive responses of using an indoor tanning bed lifetime and circumstances of using a tanning bed.

Table S16. Episodes of sunburn last summer across gender in the 2018 Health Behaviour in School-aged Children study in Ireland (*n* = 10,059)

	**Boys**	**Girls**
**Never**	29.0% (1363)	24.3% (1303)
**1 time**	25.8% (1210)	25.8% (1384)
**2 times**	21.0% (987)	23.5% (1262)
**3-4 times**	13.9% (651)	16.8% (902)
**5 times or more**	10.4% (488)	9.5% (509)

Table S17. Episodes of sunburn last summer across age in the 2018 Health Behaviour in School-aged Children study in Ireland (*n* = 10,059)

	**10-11 years**	**12-14 years**	**15-17 years**
**Never**	32.4% (567)	26.9% (1326)	22.8% (773)
**1 time**	26.8% (468)	27.0% (1328)	23.6% (798)
**2 times**	20.1% (351)	22.2% (1093)	23.8% (805)
**3-4 times**	12.2% (214)	14.0% (691)	19.1% (648)
**5 times or more**	8.5% (149)	9.9% (488)	10.6% (360)

Table S18. Episodes of sunburn last summer across social class in the 2018 Health Behaviour in School-aged Children study in Ireland (*n* = 10,059)

	**SC1-2**	**SC3-4**	**SC5-6**
**Never**	26.0% (1418)	25.8% (911)	31.5% (337)
**1 time**	26.6% (1448)	25.9% (915)	21.6% (231)
**2 times**	22.7% (1236)	22.2% (784)	21.4% (229)
**3-4 times**	15.4% (839)	16.1% (571)	13.4% (143)
**5 times or more**	9.4% (512)	10.0% (355)	12.1% (130)

Table S19. Episodes of sunburn lifetime across gender in the 2018 Health Behaviour in School-aged Children study in Ireland (n = 9992)

	**Boys**	**Girls**
**Never**	12.6% (591)	11.0% (586)
**1 time**	10.4% (489)	10.4% (553)
**2 times**	11.4% (535)	11.7% (621)
**3-4 times**	19.7% (922)	21.5% (1139)
**5 times or more**	45.8% (2145)	45.4% (2411)

Table S20. Episodes of sunburn lifetime across age in the 2018 Health Behaviour in School-aged Children study in Ireland (n = 9992)

	**10-11 years**	**12-14 years**	**15-17 years**
**Never**	14.7% (252)	12.7% (626)	8.9% (299)
**1 time**	15.7% (270)	10.6% (521)	7.5% (251)
**2 times**	13.1% (226)	12.2% (601)	9.8% (329)
**3-4 times**	21.5% (370)	20.5% (1008)	20.3% (683)
**5 times or more**	35.0% (602)	43.9% (2154)	53.5% (1800)

Table S21. Episodes of sunburn lifetime across social class in the 2018 Health Behaviour in School-aged Children study in Ireland (n = 9992)

	**SC1-2**	**SC3-4**	**SC5-6**
**Never**	11.1% (599)	11.3% (398)	16.9% (180)
**1 time**	10.0% (544)	10.7% (374)	11.7% (124)
**2 times**	11.4% (615)	11.9% (417)	11.7% (124)
**3-4 times**	21.7% (1175)	20.4% (715)	16.1% (171)
**5 times or more**	45.9% (2484)	45.8% (1607)	43.7% (465)

Table S22. Tanning bed use in the last 12 months across gender in the 2018 Health Behaviour in School-aged Children study in Ireland (*n* = 10,018)

	**Boys**	**Girls**
**Never**	97.9% (4579)	96.9% (5174)
**1 time**	0.9% (43)	1.2% (65)
**2 times**	0.4% (20)	0.6% (31)
**3-4 times**	0.2% (11)	0.5% (25)
**5 times or more**	0.5% (23)	0.9% (47)

Table S23. Tanning bed use in the last 12 months across age in the 2018 Health Behaviour in School-aged Children study in Ireland (*n* = 10,018)

	**10-11 years**	**12-14 years**	**15-17 years**
**Never**	97.7% (1680)	97.5% (4791)	96.9% (3282)
**1 time**	1.2% (20)	1.0% (48)	1.2% (40)
**2 times**	0.4% (7)	0.6% (31)	0.4% (13)
**3-4 times**	0.3% (6)	0.3% (14)	0.5% (16)
**5 times or more**	0.4% (7)	0.6% (28)	1.0% (3386)

Table S24. Tanning bed use in the last 12 months across social class in the 2018 Health Behaviour in School-aged Children study in Ireland (*n* = 10,018)

	**SC1-2**	**SC3-4**	**SC5-6**
**Never**	98.0% (5319)	96.6% (3404)	96.4% (1030)
**1 time**	0.9% (48)	1.3% (45)	1.4% (15)
**2 times**	0.3% (14)	0.9% (31)	0.6% (6)
**3-4 times**	0.3% (18)	0.4% (15)	0.3% (3)
**5 times or more**	0.5% (27)	0.8% (29)	1.3% (14)

Table S25. Tanning bed use lifetime across gender in the 2018 Health Behaviour in School-aged Children study in Ireland (*n* = 10,077)

	**Boys**	**Girls**
**Never**	97.3% (4586)	96.2% (5161)
**1 time**	0.9% (42)	1.3% (69)
**2 times**	0.4% (18)	0.6% (31)
**3-4 times**	0.6% (28)	0.7% (38)
**5 times or more**	0.8% (37)	1.2% (67

Table S26. Tanning bed use lifetime lifetime across age in the 2018 Health Behaviour in School-aged Children study in Ireland (*n* = 10,077)

	**10-11 years**	**12-14 years**	**15-17 years**
**Never**	97.2% (1695)	96.9% (4797)	96.2% (3255)
**1 time**	1.0% (18)	1.1% (53)	1.2% (40)
**2 times**	0.6% (10)	0.5% (26)	0.4% (13)
**3-4 times**	0.4% (7)	0.5% (27)	0.9% (32)
**5 times or more**	0.7% (13)	1.0% (48)	1.3% (43)

Table S27. Tanning bed use lifetime across social class in the 2018 Health Behaviour in School-aged Children study in Ireland (*n* = 10,077)

	**SC1-2**	**SC3-4**	**SC5-6**
**Never**	97.5% (5324)	95.9% (3400)	95.8% (1023)
**1 time**	1.0% (57)	1.1% (38)	1.5% (16)
**2 times**	0.4% (20)	0.7% (26)	0.3% (3)
**3-4 times**	0.5% (27)	0.8% (29)	0.9% (10)
**5 times or more**	0.6% (35)	1.5% (53)	1.0% (16)

Table S28. Having been asked about age when using an indoor tanning bed across gender in the 2018 Health Behaviour in School-aged Children study in Ireland (*n* = 280)

	**Boys**	**Girls**
**Yes, every time**	16.7% (17)	12.9% (23)
**Yes, at least once**	20.6% (21)	27.0% (48)
**No**	34.3% (35)	37.6% (67)
**Don't remember**	28.4% (29)	22.5% (40)

[a] Cleaned for a combination of 3, 4, or 5 positive responses of using an indoor tanning bed lifetime and circumstances of using a tanning bed.

Table S29. Having been asked about age when using an indoor tanning bed across age in the 2018 Health Behaviour in School-aged Children study in Ireland (*n* = 280)

	**10-11 years**	**12-14 years**	**15-17 years**
**Yes, every time**	6.8% (3)	17.6% (22)	13.5% (15)
**Yes, at least once**	25.0% (11)	24.8% (31)	24.3% (27)
**No**	36.4% (16)	29.6% (37)	44.1% (49)
**Don't remember**	31.8% (14)	28.0% (35)	18.0% (20)

[a] Cleaned for a combination of 3, 4, or 5 positive responses of using an indoor tanning bed lifetime and circumstances of using a tanning bed.

Table S30. Having been asked about age when using an indoor tanning bed across social class in the 2018 Health Behaviour in School-aged Children study in Ireland (*n* = 280)

	**SC1-2**	**SC3-4**	**SC5-6**
**Yes, every time**	10.1% (12)	19.0% (23)	12.5% (5)
**Yes, at least once**	26.9% (32)	22.3% (27)	25.0% (10)
**No**	34.5% (41)	39.7% (48)	32.5% (13)
**Don't remember**	28.6% (34)	19.0% (23)	30.0% (12)

[a] Cleaned for a combination of 3, 4, or 5 positive responses of using an indoor tanning bed lifetime and circumstances of using a tanning bed.

Table S31. Having been told to wear protective goggles when using an indoor tanning bed across gender in the 2018 Health Behaviour in School-aged Children study in Ireland (*n* = 282)

	**Boys**	**Girls**
**Yes, every time**	24.8% (26)	34.5% (61)
**Yes, at least once**	12.4% (13)	15.8% (28)
**No**	36.2% (38)	39.0% (69)
**Don't remember**	26.7% (28)	10.7% (19)

[a] Cleaned for a combination of 3, 4, or 5 positive responses of using an indoor tanning bed lifetime and circumstances of using a tanning bed.

Table S32. Having been told to wear protective goggles when using an indoor tanning bed across age in the 2018 Health Behaviour in School-aged Children study in Ireland (*n* = 282)

	**10-11 years**	**12-14 years**	**15-17 years**
**Yes, every time**	22.7% (10)	32.6% (42)	32.1% (35)
**Yes, at least once**	11.4% (5)	15.5% (20)	14.7% (16)
**No**	43.2% (19)	33.3% (43)	41.3% (45)
**Don't remember**	22.7% (10)	18.6% (24)	11.9% (13)

[a] Cleaned for a combination of 3, 4, or 5 positive responses of using an indoor tanning bed lifetime and circumstances of using a tanning bed.

Table S33. Having been told to wear protective goggles when using an indoor tanning bed across social class in the 2018 Health Behaviour in School-aged Children study in Ireland (*n* = 282)

	**SC1-2**	**SC3-4**	**SC5-6**
**Yes, every time**	33.9% (40)	28.2% (35)	30.0% (12)
**Yes, at least once**	18.6% (22)	12.9% (16)	7.5% (3)
**No**	31.4% (37)	44.4% (55)	37.5% (15)
**Don't remember**	16.1% (19)	14.5% (18)	25.0% (10)

[a] Cleaned for a combination of 3, 4, or 5 positive responses of using an indoor tanning bed lifetime and circumstances of using a tanning bed.

Table S34. Having been given advice on skin type when using an indoor tanning bed across gender in the 2018 Health Behaviour in School-aged Children study in Ireland (*n* = 287)

	**Boys**	**Girls**
**Yes, every time**	16.2% (17)	22.5% (41)
**Yes, at least once**	22.9% (24)	17.0% (31)
**No**	28.6% (30)	48.9% (89)
**Don't remember**	32.4% (34)	11.5% (21)

[a] Cleaned for a combination of 3, 4, or 5 positive responses of using an indoor tanning bed lifetime and circumstances of using a tanning bed.

Table S35. Having been given advice on skin type when using an indoor tanning bed across age in the 2018 Health Behaviour in School-aged Children study in Ireland (*n* = 287)

	**10-11 years**	**12-14 years**	**15-17 years**
**Yes, every time**	17.8% (8)	21.7% (28)	19.5% (22)
**Yes, at least once**	28.9% (13)	16.3% (21)	18.6% (21)
**No**	24.4% (11)	38.8% (50)	51.3% (58)
**Don't remember**	28.9% (13)	23.3% (30)	10.6% (12)

[a] Cleaned for a combination of 3, 4, or 5 positive responses of using an indoor tanning bed lifetime and circumstances of using a tanning bed.

Table S36. Having been given advice on skin type when using an indoor tanning bed across social class in the 2018 Health Behaviour in School-aged Children study in Ireland (*n* = 287)

	**SC1-2**	**SC3-4**	**SC5-6**
**Yes, every time**	20.7% (24)	20.8% (26)	17.1% (7)
**Yes, at least once**	19.8% (25)	21.6% (27)	9.8% (4)
**No**	40.5% (49)	39.2% (49)	51.2% (21)
**Don't remember**	19.0% (23)	18.4% (23)	22.0% (9)

[a] Cleaned for a combination of 3, 4, or 5 positive responses of using an indoor tanning bed lifetime and circumstances of using a tanning bed.

Table S37. Having been told about health risks when using an indoor tanning bed across gender in the 2018 Health Behaviour in School-aged Children study in Ireland (*n* = 286)

	**Boys**	**Girls**
**Yes, every time**	20.8% (22)	26.7% (48)
**Yes, at least once**	22.6% (24)	13.3% (24)
**No**	28.3% (30)	48.3% (87)
**Don't remember**	28.3% (30)	11.7% (21)

[a] Cleaned for a combination of 3, 4, or 5 positive responses of using an indoor tanning bed lifetime and circumstances of using a tanning bed.

Table S38. Having been told about health risks when using an indoor tanning bed across age in the 2018 Health Behaviour in School-aged Children study in Ireland (*n* = 286)

	**10-11 years**	**12-14 years**	**15-17 years**
**Yes, every time**	13.0% (6)	25.8% (33)	27.7% (31)
**Yes, at least once**	26.1% (12)	14.8% (19)	15.2% (17)
**No**	32.6% (15)	39.1% (50)	46.4% (52)
**Don't remember**	28.3% (13)	20.3% (26)	10.7% (12)

[a] Cleaned for a combination of 3, 4, or 5 positive responses of using an indoor tanning bed lifetime and circumstances of using a tanning bed.

Table S39. Having been told about health risks when using an indoor tanning bed across social class in the 2018 Health Behaviour in School-aged Children study in Ireland (*n* = 286)

	**SC1-2**	**SC3-4**	**SC5-6**
**Yes, every time**	21.5% (26)	25.8% (32)	29.3% (12)
**Yes, at least once**	17.4% (21)	16.9% (21)	14.6% (6)
**No**	40.5% (49)	41.9% (52)	39.0% (16)
**Don't remember**	20.7% (25)	15.3% (19)	17.1% (7)

[a] Cleaned for a combination of 3, 4, or 5 positive responses of using an indoor tanning bed lifetime and circumstances of using a tanning bed.
